# Underwater Undulating Propulsion Biomimetic Robots: A Review

**DOI:** 10.3390/biomimetics8030318

**Published:** 2023-07-19

**Authors:** Gongbo Li, Guijie Liu, Dingxin Leng, Xin Fang, Guanghao Li, Wenqian Wang

**Affiliations:** Department of Mechanical and Electrical Engineering, Ocean University of China, Qingdao 266000, China; li_gongbo2020@163.com (G.L.);

**Keywords:** underwater biomimetic robots, undulating propulsion robots, undulating propulsion mechanism, experimental study

## Abstract

The traditional propeller-based propulsion of underwater robots is inefficient and poorly adapted to practice. By contrast, underwater biomimetic robots show better stability and maneuverability in harsh marine environments. This is particularly true of undulating propulsion biomimetic robots. This paper classifies the existing underwater biomimetic robots and outlines their main contributions to the field. The propulsion mechanisms of underwater biomimetic undulating robots are summarized based on theoretical, numerical and experimental studies. Future perspectives on underwater biomimetic undulating robots are also presented, filling the gaps in the existing literature.

## 1. Introduction

**Figure 1:**
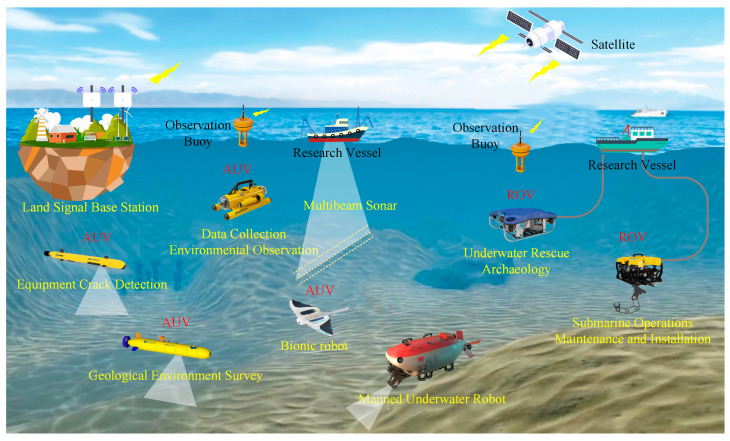
Overview of ROV and AUV application scenarios.

Underwater robots have an important role to play in future marine development and underwater activities and have broad application prospects and great potential value [1,2,3,4,5]. Researchers have designed various underwater robots for underwater missions, such as remotely operated vehicles (ROVs) and autonomous underwater vehicles (AUVs) [6,7] (Figure 1). Traditional underwater robots are composed of rigid materials and most are propelled by propellers [8,9]. However, propeller propulsion is unsuitable for implementation in harsh marine environments as it can damage aquatic life or the seabed or even become trapped in weeds [10,11]. In addition, the low efficiency and stability of propeller-driven craft in low-speed attitude adjustment conditions greatly limit the applications of propeller propulsion. Therefore, novel underwater propulsion methods are desired and are currently being investigated [7].

At present, increasing attention is being paid to bionics-based propulsion mechanisms. This propulsive method is validated in marine environments as its principle is inspired by various marine organisms. The soft bodies, good flexibility and strong environmental adaptability of marine organisms inspire new ideas for the development of underwater robots [12]. After a long period of evolution, fish have adapted to exploit the principle of hydrodynamics to move forward using tail fins or pectoral fins [13,14,15], which has become the best way to move in water [16,17]. The propulsion method employed by fish can maintain high maneuverability and change posture according to the changing environment. Inspired by the fish propulsion method, the first underwater bionic robot RoboTuna [18] was unveiled in 1994, and various types of underwater bionic robots continue to emerge. Equipped with various propulsion methods, underwater robots present positive development trends and broad application prospects [19,20,21,22,23] The term underwater biomimetic robot generally refers to robots that imitate the structure, function, or type of movement of fish species [24]. They have the basic movement characteristics of biomimetic fish and are continuously being optimized and improved on this basis. They have diverse functions, strong environmental adaptability and stability [25]. Undulating propulsion biomimetic robots are propelled by the undulating of pectoral fins, caudal fins and the body, an approach that has superior motion performance and can realize multiple motion modes [26]. In summary, undulating propulsion underwater biomimetic robots are increasingly favored by scholars due to their good maneuverability and stability. Distinct from previous reviews [7,27,28,29,30,31,32,33,34,35,36,37], this work provides a review of undulating propulsion biomimetic robots, especially the different types of propulsion mechanisms. The review is organized as follows: In Section 2, the swimming patterns of fish are described. In Section 3, an overview of existing underwater robots with undulating propulsion is presented. In Section 4, the propulsion mechanisms of undulating propulsion underwater robots are described. In Section 5, the limitations and perspectives of fluctuation-propelled underwater robots are outlined.

## 2. The Swimming Mode of Fish

Starting from the morphological function of fish, there are many swimming modes of fish in nature, the most authoritative classification of which, based on the organ of propulsion, was first proposed by Breder and Webb [38]. There are two main categories of propulsion methods, body caudal fin (BCF) propulsion and median paired fin (MPF) propulsion. In 1978, Lindsey [39] classified the different families of fish species according to their mode of propulsion, which provided the essential foundation for the improvement of the overall performance of undulating propulsion biomimetic robots.

**Figure 2:**
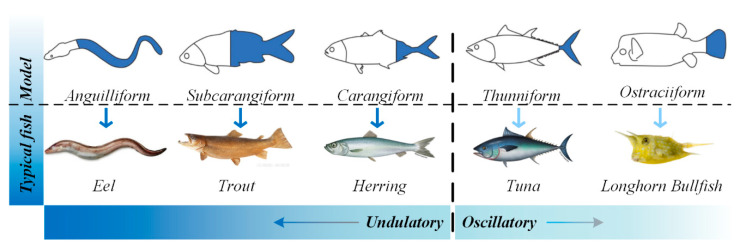
BCF propulsion mode [29].

Figure 2 shows the fish species that employ BCF mode propulsion [32]. For this propulsion mode, the main body types are anguilliform, subcarangiform, carangiform, thunniform and ostraciiform, and the representative fish species for these body types are the eel, trout, herring, tuna and longhorn cowfish, respectively. Fish employing BCF mode propulsion gradually transition from undulating to oscillatory propulsion according to their different driving methods, as shown by the dashed line in Figure 2. In the figure, the first three propulsion modes are undulating and the last two are oscillatory. BCF propulsion modes are characterized by a high swimming speed—swordfish can reach a speed of 110 km/h—and good energy efficiency—tuna can maintain 90% energy efficiency while swimming for a long time [40]. Fish with the anguilliform body type employ fluctuations of the whole body and tail fin. This type of fish has a slender body and a faster swimming speed. Subcarangiform and carangiform fish species are relatively similar; the difference is that the former uses 1/2 of their body length for fluctuations, while the latter uses only about 1/3 of their body length for propulsion [41,42]. Compared with the anguilliform model, the fluctuation range is small, but the caudal fluctuation frequency is high. The propulsion of thunniform fish comes from the tail fin [43]. Based on hydrodynamic models, thunniform propulsion has been estimated to produce mechanical efficiencies in the range of 80–90% [18,44,45]. The fastest fish (tuna, dolphin) use this propulsion mode [46,47,48,49]. Fish species with the ostraciiform body type adopt an oscillatory propulsion mode. This type of fish simply oscillates the caudal fin, which induces only a small thrust; thus, the speed of travel is relatively slow [50].

**Figure 3:**
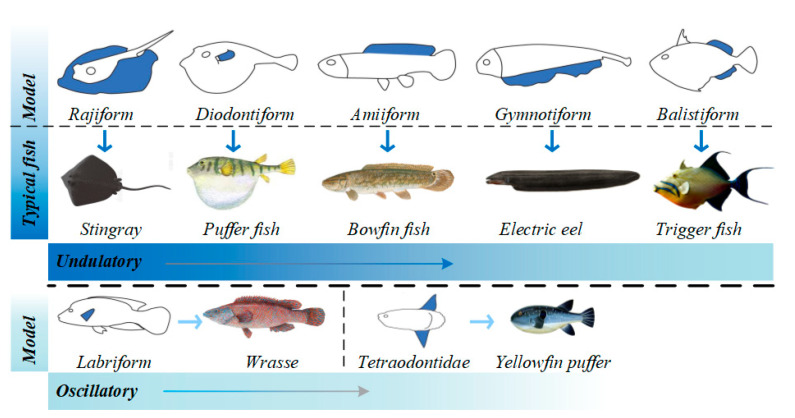
MPF propulsion mode [29].

Figure 3 shows the fish that employ MPF mode propulsion [32]. The main body types seen are rajiform, diofontiform, amiiform, gymnotiform, balistiform tetraodontidae and labriform, and the corresponding representative fish species are the stingray, puffer fish, bowfin fish triggerfish, yellowfin puffer and wrasse, respectively. The first five body types employ undulating propulsion, and the latter two employ oscillatory propulsion. As shown by the dotted line in Figure 3, most of the fish that employ MPF mode propulsion exhibit undulating propulsion. Rajiform fish species have soft and wide pectoral fins in a triangular shape and use the large fluctuations of the pectoral fins to generate driving power [51]. The most representative diodontiform fish species is the puffer fish. The puffer fish utilizes fluctuation of the pectoral fins on both sides of its body to propel [52]. Compared with rajiform fish species, the pectoral fins of diodontiform fish are smaller; thus, the traveling speed is slower. Amiiform fish generally have very long dorsal fins, which leads to larger amplitude waves during undulations, enabling larger movements [53]. Gymnotiform fish are similar to amiiform fish, except that their propelling fins are on the lower side of their bodies, and the power to move is generated through the oscillatory movement of the anal fin [54]. Balistiform fish generate the driving force using their dorsal and anal fins [55]. Balistiform-based locomotion has a very high propulsive efficiency compared to BCF propulsion modes. And compared to propulsion arising from the undulation of a slender fin, balistiform-based locomotion allows more maneuverability [56].

Overall, it appears that BCF-based propulsion permits a higher travel speed than MPF-based propulsion, and uses the rapid oscillation of wings to obtain efficient propulsion [56]. For example, in terms of BCF mode, the average swimming speed of Chinook salmon is 0.6 m/s, and their maximum swimming speed can exceed 6 m/s [57]. The swordfish, with its carangiform body type, has a swimming speed close to 2 m/s, and its maximum speed can even reach 27 m/s [58]. Conversely, with MPF mode, the average swimming speed of the fish is relatively slow. For example, the average swimming speed of the common stingray is 0.81 m/s, and its maximum speed is only 13 m/s [59]. It can be seen that most of the fish employing BCF-based propulsion swim faster than fish employing MPF-based propulsion. Different fish species have different morphological functions, which guarantee their survival. Researchers should study the morphological functions of fish species for use as the inspiration for bionics-based propulsion systems.

## 3. Classification of Undulating Propulsion Underwater Robots

Inspired by various morphological functions of fish species, underwater biomimetic robots have been designed and developed. On the basis of inheriting the characteristics of biomimetic objects, different materials and structures have been applied to optimize biomimetic robots. In this section, according to the requirements of application scenarios, the motion performance and travel efficiency of underwater biomimetic robots are highlighted. The corresponding underwater biomimetic robots are classified according to the biometric fish model that serves as their inspiration, and recent developments are also briefly presented.

### 3.1. Anguilliform Biomimetic Robots

Anguilliform biomimetic robot fish, mainly inspired by the soft eel, have flexible joints and fluctuate their flexible bodies and tail fins to produce motion [60]. The AmphiBot II, which was designed by Alessandro Crespi, featured a maximum torque that was 3.5 times higher than that of the earlier AmphiBot I, which greatly enhanced its propulsion efficiency [61]. Salamandra Robotica II can crawl and swim both on land and in water. To further enhance the working capabilities of such robots, the Lampetra Project robot was proposed, which not only had muscle-like execution capabilities but also can work continuously for 5 h [62]. With the gradual maturation of the biomimetic robot design concept, the modular research method was applied in the Mamba Waterproof Snake Robot [63] and Multi-joint Underwater Robot [64], which not only facilitated the subsequent upgrading of the robots but also improved the propulsion efficiency. Details of the above-mentioned designs are presented in Table 1.

### 3.2. Subcarangiform and Carangiform Biomimetic Robots

Subcarangiform and carangiform fish have similar movement patterns. Robots with these forms have high undulating propulsion efficiency, fast swimming speed, and high application value, and hence, numerous studies have been conducted, as shown in Table 2. The G9fish was a relatively mature subcarangiform robotic fish [67]. Its design was derived from a set of mature underwater biomimetic robots motion modeling methods and it could realize two traveling modes, straight−line cruise and C−shaped sharp turn. Relying on mature motion modeling methods, the number of motion modes of biomimetic robots has constantly increased. Hydraulic Soft Robotic Fish could realize diving swimming and the Wire−Driven Robot Shark could realize ascending swimming and position−maintaining actions [68]. In addition, the Soft−Bodied Robotic Fish [69] could simulate escape response maneuvers and the Fabricated Bionic Robotic Fish [70] could actively avoid obstacles and quickly determine the shortest path to a target point. To further improve the kinematic performance of underwater robots, some scholars have focused their research on innovations in biomimetic materials. Compared with biomimetic robots with rigid structures such as ISplash−I [71], ISplash−II [72] and the Four−link Robotic Fish [73], the Biomimetic Fish Robot [74] used piezoelectric composites for the robot’s muscle structure. The Fabricated Bionic Robotic Fish utilized spring−based shape memory alloys as propulsion mechanisms, which not only improved its kinematic performance but also greatly enhanced its maneuverability [70].

### 3.3. Thunniform and Ostraciiform Biomimetic Robots

Thunniform and ostraciiform fish employ the oscillatory propulsion mode. Their traveling speed is fast; however, their mobility is inferior. There have been relatively few studies of this type of motion due to the limited number of application scenarios [82]. As shown in Table 3, the Miniature Robotic Fish took live fish as the biometric research object, studying the interaction between robotic fish and live fish to provide a basis for the study of fish habits [83]. The Gliding Robotic Dolphin combined the advantages of dolphins and underwater gliders, which not only increased the speed of the robots but also enabled the smooth gliding motion and attitude adjustment of the robot [84]. Although the Single−Motor−Actuated Robotic Fish [85] and Thunniform Robotic Fish [43] had a simple structure, they performed well in terms of motion and maneuverability.

Ostraciiform biomimetic robots have three propulsion modules, a tail fin and two pectoral fins. With the help of the CPG control network, the BoxyBot could swim in water and crawl on the ground [86]. The Boxfish−like Robot could swim in three dimensions and used a rolling motion for attitude control, which greatly enhanced its motion performance [87]. Details of the above−mentioned designs are provided in Table 4.

**Table 3:** Thunniform biomimetic robots.

Robot	Date	Description	Main Contributions	Picture
Single−Motor−Actuated Robotic Fish [85]	2016	⬤ Fewer joints ⬤ Mechanical design, motion analysis and attitude control ⬤ Controlled by a single motor	➢ Relies on simple mechanical structure and motion control to improve the overall motion performance	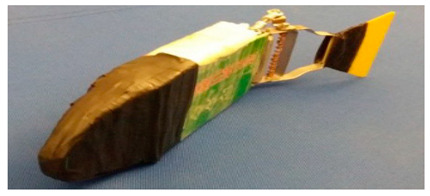
Gliding Robotic Dolphin [84]	2015	⬤ Combines the advantages of dolphins and underwater gliders ⬤ Gliding motions, dolphin−style double−jointed flapping swimming, and stable chest propulsion motions	➢ Rear−drive propulsion and fluke can realize dolphin−like fast swimming ➢ Uses pectoral fins and fluke to realize attitude adjustment and smooth glide	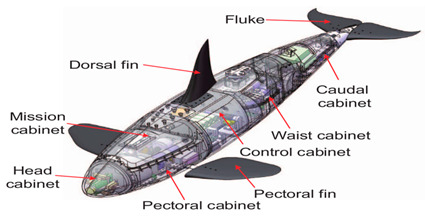
Thunniform Robotic Fish [43]	2022	⬤ An elastic chord and a tail fin ⬤ Controlled by two motors ⬤ The tail fin part can provide a fixed amplitude	➢ Relationship between caudal fin frequency, oscillation amplitude and travel speed	N/A
Mackerel Robot [88]	2013	⬤ The power, wakefield and propulsion speed of the robotic fish can be measured simultaneously	➢ Quantitative measurement of robotic fish propulsion efficiency achieved	N/A

**Table 4:** Ostraciiform biomimetic robots.

Robot	Date	Description	Main Contributions	Picture
Boxfish Robot [86]	2017	⬤ With three micro−servo motors ⬤ Speed, thrust and hydrodynamic aspects ⬤ Three types of pectoral fins	➢ The combination of a quarter−circle pectoral fin and a triangular tail fin provides the fastest speed	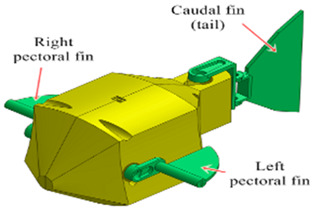
Boxfish−like Robot [87]	2013	⬤ Multiple 3D swimming modes and roll attitude control ⬤ CPG control network ⬤ Inertial measurement unit	➢ CPG closed−loop control for robotic fish yaw and roll	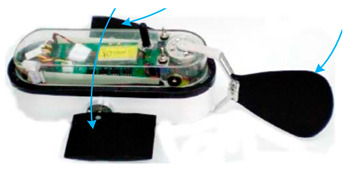
BoxyBot [89]	2007	⬤ Non−steady−state swimming and crawling ⬤ Three actuated fins ⬤ Swim in water and crawl on solid ground	➢ The CPG model can generate fin trajectories online ➢ CPG change control parameters	N/A

### 3.4. Rajiform Biomimetic Robots

Inspired by rajiform fish, some biomimetic robot fish employing the MPF propulsion modes have been proposed, as listed in Table 5. The skate is a typical rajiform with a flat body, wide pectoral fins and large fluctuating motions. Inspired by the skate’s soft body skeleton, the Self−Powered Soft Robot developed by Zhejiang University had high application value and consisted of a full−body soft structure without any rigid structure [90]. In addition, it used a dielectric elastomer material for its flutter wings, with electronic components integrated into a silicone body, and was successfully launched in a field test in the Marianas Trench down to a depth of 10,900 m. Other rajiform underwater biomimetic robots also demonstrated good kinematic performance, such as the IPMC Manta Ray [91], the Manta Ray Robot [92] and the Bionic Fin Manta Ray [93]. The difference between these three robots is their material implementation, that is, IPMC Manta Ray was made of ionic polymer metal composite materials, the Manta Ray Robot was made of three flexible materials, and the Bionic Fin Manta Ray was made of flexible PVC material. Both the Soft Body Single−Dual Actuator Ray [94] and the Cartilage Structure Underwater Robot [95] were inspired by stingrays, with the former being made of silicon−based cartilage and the latter being made of steel.

### 3.5. Amiiform Biomimetic Robots

The Amiiform differs from the gymnotiform in that its undulating propulsion mechanism is a dorsal fin. As shown in Table 6, RoboGnilos [98] and the Bio−inspired Amphibious Robot [99] were inspired by Gmnarchus Niloticus. The former had an undulating dorsal fin above the body and the latter had undulating fins symmetrically distributed on both sides of the body.

### 3.6. Gymnotiform Biomimetic Robots

Gymnotiform and anguilliform fish are similar, but gymnotiform fish are propelled by undulating ventral and anal fins, while anguilliform fish are propelled by undulating body and caudal fins. As shown in Table 7, most of the gymnotiform biomimetic robots were inspired by the black ghost knife fish, such as the NKF−II [101], Gymnotiform Undulating Fin Robot [102] and Undulatory Fin Propulsion Bio−Inspired Robot [103]. The Robotic Knifefis was inspired by the South American electric knife fish, and its key drive parameters were investigated for the undulating propulsion of the ribbon fin [104].

### 3.7. Labriform Biomimetic Robots

Labriform biomimetic robotic fish employ the oscillatory propulsion mode, as shown in Table 8. Related underwater biomimetic robots use the oscillating travel of the pectoral and caudal fins, but to date, there have been few studies published. The Pectoral Fin and Dual Caudal Fin Robot used the tail fin as the main propeller and was capable of free swimming and active obstacle avoidance [106]. The Flexible Pectoral Fin Joint Labriform Robot used the rigid pectoral fin as the main propulsion structure, which was connected by flexible feather joints to enhance motion performance [107].

## 4. Undulating Propulsion Underwater Robot Propulsion Mechanisms

Exploring the propulsion mechanisms of underwater biomimetic robots is essential for the development of new prototypes and the continuous refinement of existing types. Researchers have investigated propulsion mechanisms by three main methods: theoretical analysis, numerical simulation and experimental testing. The main findings are presented below.

### 4.1. Theoretical and Numerical Simulation Studies

British scholar James Gray observed the daily food intake and swimming distance of dolphins in 1936 and found that the work done by dolphins swimming was seven times the energy provided by the food they ate, which was called ‘Gray’s problem’ [109]. Gray pioneered the study of biomimetic fish. In 1952, the British physicist Geoffrey Taylor proposed an analytical model based on the resistance theory [110]. He used the ‘static fluid theory’ to solve the dynamics of fish movement. The theory considered the influence of viscous force but the effect of fluid inertial force was ignored, and hence, it was only suitable for small Reynolds number conditions. In 1955, Hancock [109] built on this achievement and put forward the ‘large−scale resistance theory’; however, the effect of fluid inertial force was still ignored. In 1960, Lighthill [111] derived an initial mathematical model of the propulsion mechanism of trevally fish; inspired by the ‘slender body theory’, he successfully applied the theory to the hydrodynamic analysis of fish swimming and then, in 1970, proposed the ‘large−oscillatory slender body theory’ according to the oscillatory amplitude of fish fins [112]. In 1960, Wu et al. [113,114,115] proposed the ‘two−dimensional undulating plate theory’, in which the research object was simplified into a flexible two−dimensional thin plate with zero thickness and simultaneously considered the inertia, leading−edge suction and wake spread. In 1971, based on the theory of two−dimensional plate fluctuations, the ‘unsteady two−dimensional undulating plate theory’ was proposed, and it was successfully used to analyze the motion performance of flat crescent−shaped fishes. In 1977, Chopra [116] studied the changes in shape of fish fins of different shapes when swimming and developed the ‘two−dimensional resistance theory’, which was considered to consist of the ‘large−oscillatory resistance theory’ and the ‘large−oscillatory slender body theory’. In 1991, Cheng et al. [117] simplified the fish model into a three−dimensional elastic plate and proposed the ‘three−dimensional undulating plate theory’.

With the application of the digital particle imaging tester (DPIT) to the research of fish propulsion mechanisms, studies have become focused on the relationship between eddy currents and fish swimming [118]. In 1994, Stamhuis [119] first applied DPIT technology to study live fish and analyze the changes in the surrounding flow field when the fish swam. Triantfyllou [120] observed that jets appeared behind the body when fish swam, and put forward the ‘jet propulsion theory’. Recently, with the mature application of simulation software and fluid dynamics software, CFD is used increasingly for kinematics and dynamics analysis [121].

**Figure 4:**
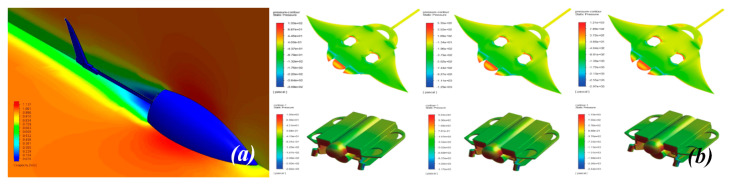
Simulations of some biomimetic robotic fish. (**a**) Thunniform Robotic Fish [43] (**b**) Manta Ray Robot [92].

Wai Pik Lau [122] derived a propulsion model for underwater biomimetic robots based on elongated body theory and created a simulation model for kinematic analysis. The Mackerel Robot designed by Li Wen used this simulation model to analyze the relationship between amplitude, efficiency and propulsion speed [88]. As shown in Figure 4, a simulation of the kinematic body drag distribution of a robot fish was carried out by Iliya Mitin et al. [43]. Finite element analysis of the kinematics and hydrodynamics of a robot fish was carried out by Qimeng Liu et al. [92].

There is still a certain gap between theoretical derivation, numerical simulation results and actual testing of robot fish due to factors such as the material, structure, and operating environment of underwater biomimetic robots. Although simulation results can be utilized as a reference for mechanism exploration, it is still of critical importance to carry out an experimental study of the prototype.

### 4.2. Experimental Studies on the Propulsion Mechanism

#### 4.2.1. Experimental Studies of Anguilliform Robots

As shown in Table 9, the Multi−joint Underwater Robot was tested in a river and the curves of lateral motion and longitudinal motion were obtained. The overall motion curve was smooth, which verified the good motion performance of the robot in the river [64]. The soft eel robot was tested in a water tank in the laboratory. With the help of high−speed cameras, it was found that swimming efficiency was dependent on both body fluctuations and body posture in situ [65]. The Lampetra Project has conducted experiments both in a laboratory tank and on land. They found that the robot was able to autonomously avoid different obstacles and swim continuously at the speed of a real creature for 5 h. The robot has a length of 0.99 m and a velocity of 0.3 m/s. For an anguilliform robot, this is a relatively high swimming speed [62]. Experiments on Salamandra Robotica II also showed that changing the curvature of the body can control the trajectory of the robot [66].

#### 4.2.2. Experimental Studies of Subcarangiform and Carangiform Robots

As shown in Table 10, G9fish demonstrated that biomimetic modeling with the approximation function was feasible. It has a length of 0.52 m and a speed of 0.2 m/s. [67]. Experiments with the Biomimetic Fish Robot showed that the shape and oscillating frequency of the tail fin directly affected the swimming speed, and the robot reached the highest swimming speed at the natural frequency of the driving tail system [74]. Experiments with the ACP Robot Fish proved that materials used in soft biomimetic robots needed to have different bending moments [81]. The Hydraulic Soft Robotic Fish was a flexible biomimetic robot that could achieve continuous body deformation in water, and it relied on the manufacture of soft drives, utilizing the creation of arbitrary fluid passages to achieve a wide range of continuous curved profiles [68]. The parameters of the Fabricated Bionic Robotic Fish [70], ISplash-I [71] and ISplash-II [72] were verified through experiments, and their speed in the tank was close to that of real fish, showing good motion performance. In particular, ISplash-I can reach 0.85 m/s, which is a relatively high speed [71]. According to experiments with the CPG-based Biomimetic Robotic Fish, the forward speed of the robot increased with the oscillating amplitude and frequency of the body [123].

#### 4.2.3. Experimental Studies of Thunniform Robots

As shown in Table 11, researchers analyzed the motion speed of Thunniform Robotic Fish and the mechanical characteristics of the tail fin and it was found that speed increases with increasing vibration frequency of the tail fin [43]. The vortex field behind the Miniature Robotic Fish’s movement and on both sides of its body was analyzed and it was found that non-static water flow would attract live fish [83]. The relationship between the amplitude, efficiency and travel speed of the Mackerel Robot was studied to determine the optimal propulsion efficiency of the robot. It has a length of 0.588 m and a speed of 0.3 m/s [88]. The Single-Motor-Actuated Robotic Fish has a length of 0.37 m and a speed of 1.14 m/s, and, thus, it travels relatively fast [85].

#### 4.2.4. Experimental Studies of Rajiform Robots

As shown in Table 12, Self-Powered Soft Robots achieved good results in physical tests. Relevant experimental researches were not only completed in lakes but also at a depth of 10,900 m in the Mariana Trench, and the robot swam autonomously in the deep sea at 3224 m in the South China Sea. Thus, experiments proved that underwater biomimetic robots can work in the deep sea, a result that has high application value [90]. Experimental tests of the Cartilage Structure Underwater Robot were similarly successful, although only in laboratory tanks, and the cartilage structure showed extremely high undulating propulsion efficiency [95]. The Manta Ray Robot achieved simple pitch and roll motion patterns in the tank, very close to the motion performance of live fish [92]. The swimming speed of IPMC Manta Ray is 0.067 BL/s, and the mobile power consumption is below 2.5 W [91].

#### 4.2.5. Experimental Studies of Amiiform and Gymnotiform Robots

In the RoboGnilos undulating propulsion experiment, inclined fin rays produced higher speed and efficiency than vertical fin rays. The Bio-inspired Amphibious Robot could realize three-dimensional swimming in the water and could crawl on the ground [99]. Its motion frequency was 0.5 Hz to 2.5 Hz, and the speed and thrust increased with frequency both underwater and on the ground. The Undulatory Fin Propulsion Bio-Inspired Robot was able to perform various swimming movements like a live fish, and the swimming efficiency was studied [103]. Details of experimental studies of amiiform and gymnotiform robots are presented in Table 13 and Table 14.

## 5. Limitations and Future Perspectives

The use of undulating propulsion for underwater robots plays a significant role in harsh marine environments. Although there have been many achievements, there are still some limitations in terms of motion performance, energy utilization and environmental perception. Firstly, the motion performance and maneuverability of undulating propulsion underwater biomimetic robots still need to be enhanced. Recent studies have mainly been conducted in stable fluid environments and how to deal with the impact of harsh water environments still needs to be supplemented and improved. Secondly, improving the energy utilization of underwater biomimetic robots is required for engineering applications, especially solving the energy consumption problem of long-term motion. Thirdly, how to improve robotic perception is the key to expanding the application of robots. Biomimetic robots usually rely on a few simple sensors to perceive the surrounding environment, but they cannot perceive the surrounding environment in harsh conditions as real fish can. Research on undulating propulsion underwater biomimetic robots is still in its infancy and needs continuous optimization in many aspects.

With the further development of biomimetics and material science, underwater biomimetic robots will achieve breakthroughs. Undulating propulsion underwater biomimetic robots will also have new prospects. These prospects include: (1) Given the complexity of the operating environment and tasks, future underwater biomimetic robots will need to not only possess the ability to swim but also possess other abilities, such as underwater grasping, sampling, transport, etc. (2) Increased biological similarity: underwater biomimetic robots will be closer in shape and movement to biological fish, and the degree of simulation and biological similarity will be continuously improved. (3) Improvement of movement efficiency and operation efficiency: with the improvement in control methods and battery technology, it is believed that future underwater biomimetic robots will have higher movement efficiency and operation efficiency. To sum up, future undulating propulsion underwater biomimetic robots will get closer to the form and function of natural organisms, and their motion performance and operating ability will also be continuously enhanced. At the same time, they will have a wider range of applications, including underwater exploration, underwater rescue, submarine engineering, underwater archaeology and aquarium-based entertainment.

## 6. Conclusions

Underwater biomimetic robots have strong operability, stability and mobility in the complex marine environment, all of which play a significant role in underwater exploration, rescue, mining, maintenance and scientific research. This paper provides a brief introduction to fish-based biometric propulsion. Existing underwater biomimetic robots are classified and the main achievements in this field are outlined, as well as the characteristics and advantages of each type. The main propulsion mechanisms of current underwater biomimetic robots are presented mainly through the content of experimental studies, emphasizing the importance of experimental studies to the discipline. The limitations and prospects of underwater biomimetic robots are also outlined, demonstrating that, in the future, underwater biomimetic robots will be developed with multiple functionalities and high bio-similarity and operational efficiency.

## Figures and Tables

**Table 1:** Anguilliform biomimetic robots.

Robot	Date	Description	Main Contributions	Picture
Multi-Joint Underwater Robot [64]	2022	⬤ Modular design ⬤ Control system with 4 motion models ⬤ With controllable propellers	➢ First-generation prototype ➢ Kinematic and dynamical models	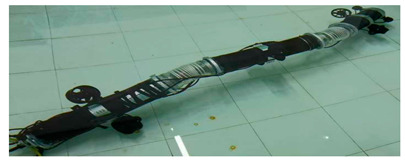
Soft Eel Robot [65]	2022	⬤ 4 pairs of soft actuators ⬤ The maximum speed reaches 19 cm/s	➢ The frequency of robot muscle unit (0.83~1.67) Hz	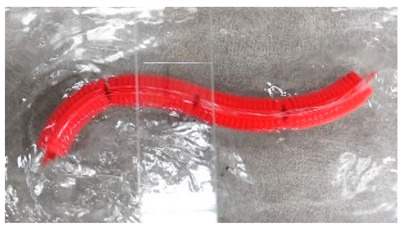
Mamba Waterproof Snake Robot [63]	2014	⬤ Modularization and Reconfigurability ⬤ With a torque sensor in each joint	➢ Completion of the design based on strain gages	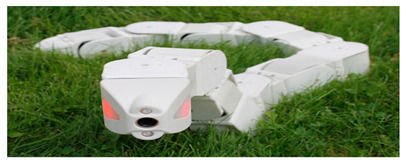
Salamandra Robotica II [66]	2013	⬤ Walk and swim ⬤ 4 legs and a driven spine ⬤ Only a single plane of motion	➢ Salamander-like robots ➢ The movement patterns of salamander-like robots	N/A

**Table 2:** Subcarangiform and carangiform biomimetic robots.

Robot	Date	Description	Main Contributions	Picture
Untethered High−Performance Robotic Tuna [75]	2022	⬤ Inspired by tuna ⬤ Through mechanism optimization and steering strategies design	➢ Both high swimming speed and steering maneuverability ➢ Novel design of redundant joints to increase the number of swimming patterns	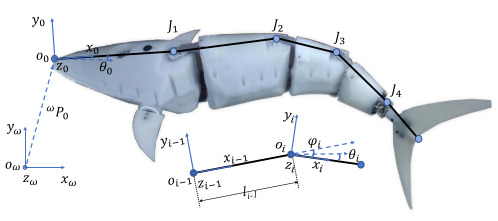
Untethered Bioinspired Robotic fish [76]	2022	⬤ With high−frequency oscillation and a compliant passive mechanism	➢ An actuation system with a powerful output and a compact structure	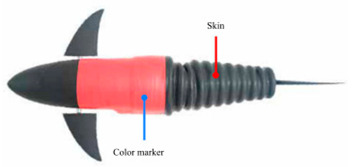
Bio−inspired AUV [77]	2021	⬤ Inspired by a pink salmon ⬤ With numerical investigation results	➢ These hydrodynamic results are utilized to adapt a fish−like aquatic unmanned vehicle from conceptual design to working prototype	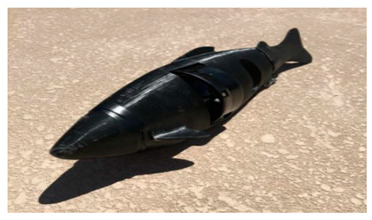
Four−link Carangiform Fish Robot [78]	2016	⬤ With a flexible multi−joint propulsion mechanism ⬤ Serve as the spine system of the fish body	➢ Move forward freely, avoid obstacles, and quickly find the shortest path to the target	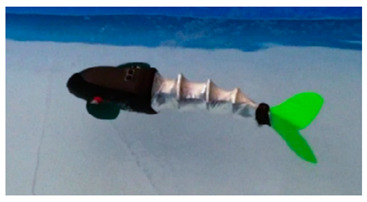
Four−link Robotic Fish Large Pectoral Fin Control Surfaces [73]	2014	⬤ Multiple artificial control surfaces ⬤ Embedded vision system ⬤ Relies on the caudal fin and the secondary fins for propulsion	➢ Equipped with a monocular visual recognition system ➢ Autonomous obstacle avoidance	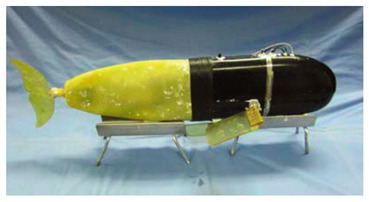
Soft−bodied Robotic Fish [69]	2013	⬤ An autonomous soft robot ⬤ Quick escape response ⬤ An array of fluidic elastomer actuators	➢ Soft robots display independent, fast body movements like biological fish	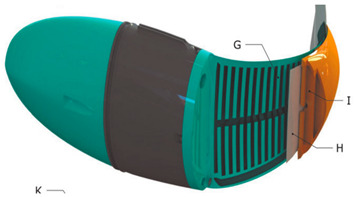
Self−contained Hydraulic Fish [79]	2013	⬤ Soft fluidic elastomer robot ⬤ Three viable actuator morphologies ⬤ Internal channel structure	➢ Provided a new actuator design and manufacturing method	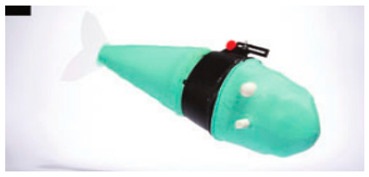
Improved ACP Robot Fish [80]	2012	⬤ A flexible caudal fin to provide thrust ⬤ Rigid head, articulated torso, and compliant caudal fin ⬤ With flow and pressure microelectromechanical sensors	➢ Utilizing low−cost MEMS sensor ➢ Quantify the normal force of the propulsion element	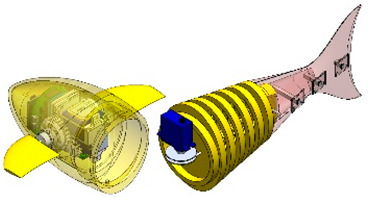
G9fish [67]	2010	⬤ A new multi−joint robot fish swimming motion modeling method ⬤ An improved trajectory approximation ⬤ Tail with servo motors	➢ An error function for digital approximation ➢ A lookup table for online optimization	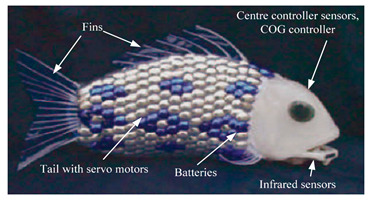
Fabricated Bionic Robotic Fish [70]	2021	⬤ Spring−based shape memory alloys ⬤ Driven by a shape memory alloy (SMA) spring actuator	➢ Complete the undulating march of subcarangiform fish ➢ Can realize the two−way shape memory effect	N/A
ACP Robot Fish [81]	2018	⬤ Based on the Lighthill fish swimming model ⬤ Obtained the distribution law of thrust	➢ A manufacturing method of the biomimetic robot fish from theoretical model	N/A

**Table 5:** Rajiform biomimetic robots.

Robot	Date	Description	Main Contributions	Picture
Cartilage Structure Underwater Robot [95]	2021	⬤ Inspired by stingrays ⬤ Silicon−based cartilage and soft tissue	➢ Cartilage structure can improve efficiency ➢ The stiffness gradient is important for locomotion performance	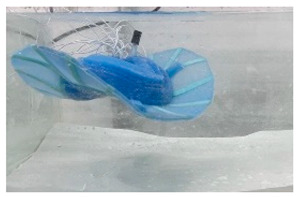
Manta Ray Robot [92]	2021	⬤ Inspired by the manta ray ⬤ Modular design ⬤ The flapping wing consists of three different soft materials	➢ Simple pitching and rolling and continuous and stable swimming ➢ As the frequency increases, the swimming speed increases	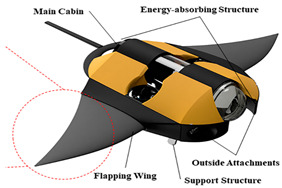
Hybrid Manta Ray Robot [96]	2021	⬤ Driven by two pectoral fins and two vertical propellers ⬤ The hydrodynamics of the torso are analyzed	➢ Combined with Fluid mechanics, the 6−DOF motion mode of the robot is analyzed	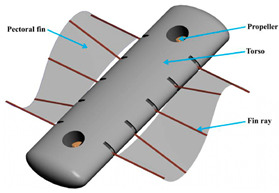
RoMan−II [97]	2012	⬤ Multi−fin thruster based on CPG ⬤ With flexible membrane propulsion ⬤ Motors distributed on both sides	➢ Determined the framework of the CPG control method	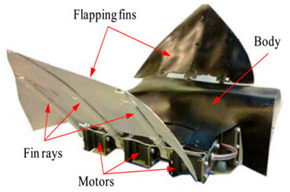
Self−powered Soft Robot [90]	2021	⬤ Inspired by deep−sea snails ⬤ A high−pressure−resistant soft robot ⬤ With dielectric elastomer materials and electronic components	➢ The whole body is soft ➢ Can swim in deep seas ➢ The electronic components and soft actuators have good pressure resilience	N/A
Bionic Fin Manta Ray [93]	2015	⬤ Inspired by the manta ray ⬤ Passive flexibility of the pectoral fins ⬤ The servo motor actively oscillates	➢ Passive flexible pectoral fins ➢ A simple and efficient propulsion structure	N/A
IPMC Manta Ray [91]	2012	⬤ Ionomer metal composites ⬤ IPMC as artificial muscles ⬤ Equipped with a lightweight, compact Li−ion polymer battery	➢ The pectoral fins can produce up to 100% tip deflection and 40° twist	N/A

**Table 6:** Amiiform biomimetic robots.

Robot	Date	Description	Main Contributions	Picture
Bio−inspired Amphibious Robot [99]	2021	⬤ Inspired by Gymnarchus Niloticus ⬤ The undulating fins are placed on both sides ⬤ Swimming and crawling brushless motor−driven rigid fin rays	➢ Mechanical method to create fins by applying force to pristine flexible membranes achieved	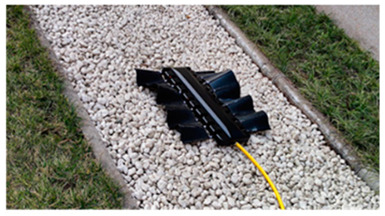
RoboGnilos [98]	2009	⬤ Inspired by Gymnarchus Niloticus ⬤ Modular independent motors ⬤ A multi−joint dynamic system	➢ Establishing a qualitative relationship between dynamical performance and undulating parameters	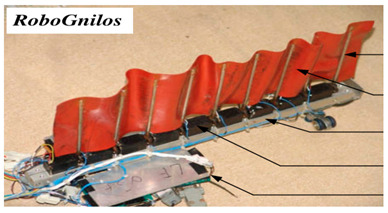
Dorsal Undulation Fin Robot [100]	2016	⬤ Two kinds of biomimetic undulating fins ⬤ Two kinds of propulsion devices ⬤ Fixed undulating and independent drive mode	➢ Prototypes guided by dynamic and kinematic models	N/A

**Table 7:** Gymnotiform biomimetic robots.

Robot	Date	Description	Main Contributions	Picture
Undulatory Fin Propulsion Bio−Inspired Robot [103]	2018	⬤ Forward swimming, reverse movement, diving, maintaining a position and vertical swimming ⬤ Equipped with 16 DC motors	➢ Fin kinematics, hydrodynamics and thrust generation of undulating fin propulsion	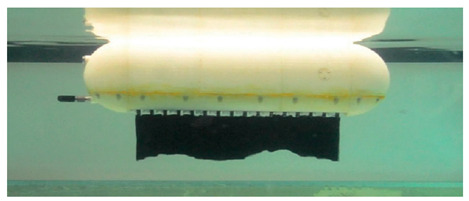
Gymnotiform Undulating Fin Robot [102]	2018	⬤ Inspired by the South American black ghost knife fish ⬤ A polyester film surface and DC motor	➢ Equations of force generated by continuous and discontinuous sine waves	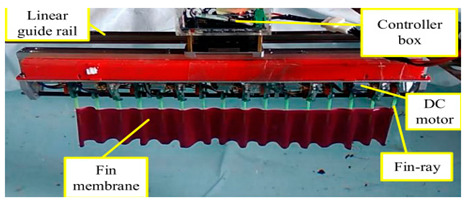
Knifefish Robot [105]	2011	⬤ Inspired by knife fish ⬤ With 6 servo motors ⬤ The fins are operated by wireless remote control	➢ The influencing factors of fin propulsion efficiency and thrust	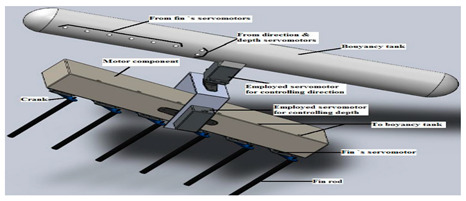
Robotic Knifefis [104]	2011	⬤ Inspired by South American electric knife fish ⬤ A custom−printed circuit board	➢ Focus on the key driving parameters of ribbon fin propulsion	N/A

**Table 8:** Labriform biomimetic robots.

Robot	Date	Description	Main Contributions	Picture
Wrasse Robot [108]	2009	⬤ Double pectoral fins ⬤ Each actuator is differentially controlled	➢ Higher flapping frequency does not necessarily result in higher swimming speed	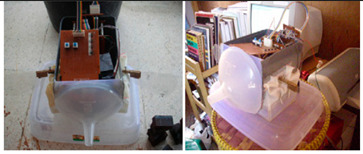
Pectoral Fin and Dual Caudal Fin Robot [106]	2016	⬤ Inspired by insect wings and fish fins ⬤ Two tail fins ⬤ Two pectoral fins	➢ Multifunctional maneuvering motion, motion switching and obstacle avoidance	N/A

**Table 9:** Anguilliform biomimetic robots: experimental research.

Robot	Test Objectives	Test Conclusions	Picture
Multi-Joint Underwater Robot [64]	⬤ Obtained the horizontal motion and vertical motion curves	➢ Overall motion control results are satisfactory	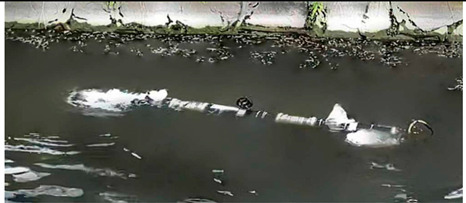
Soft Eel Robot [65]	⬤ Images of frequency and motion posture were obtained	➢ Swimming efficiency depends both on thrust generated and on in situ body position	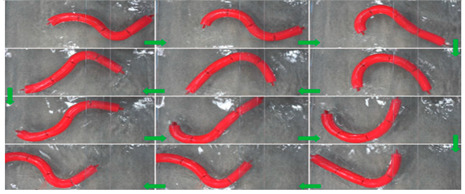
Salamandra Robotica II [66]	⬤ Analyzed the relationship between attitude and speed	➢ The speed increases monotonously with the frequency ➢ Changing the curvature of the body can control the trajectory	N/A

**Table 10:** Anguilliform biomimetic robots: experimental research.

Robot	Test Objectives	Test Conclusions	Picture
Untethered High-Performance Robotic Tuna [75]	◆ Speed Performance ◆ Steering Performance	➢ Can achieve both high swimming speed (2.26 m/s) and steering maneuverability with 0.48 BL (body lengths) turning radius	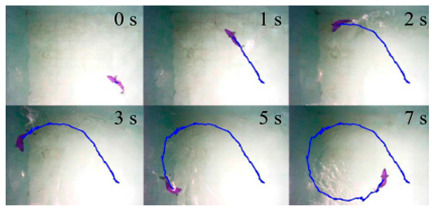
Untethered Bioinspired Robotic Fish [76]	◆ Free-swimming and dynamic model validation ◆ Pitch maneuvers ◆ Leaping motion tests	➢ Speed of 3.8 BL/s ➢ Achieves high pitch maneuvers by performing an agile front flip motion with a radius of 0.4 BL and an average angular velocity of 439°/s	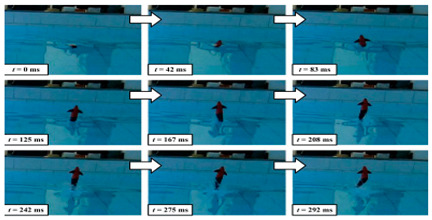
G9fish [67]	◆ Two traveling modes, cruising straight line and C-shaped sharp turn	➢ The relative tail motion and approximation error functions have good performance	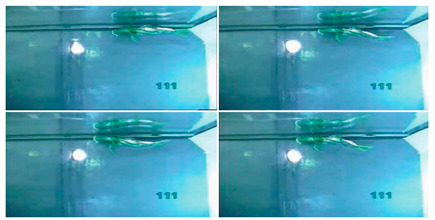
Bio-inspired AUV [77]	◆ The performance of the motor and AUV was tested	➢ Accomplishes turns at a body-length-to-turn-radius ratio of 1:1, at a swimimg velocity of 1.5 BL/s	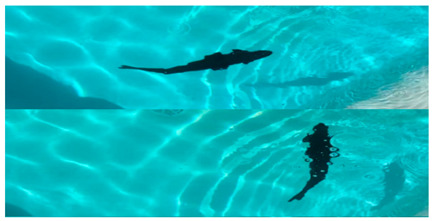
Soft-bodied Robotic Fish [69]	◆ A simulated escape response exercise ◆ Compared the kinematics and controllability	➢ Agonistic duration has strong authority over escape angle and minimal authority over linear escape velocity	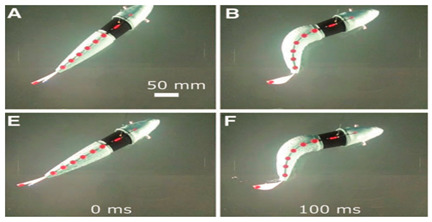
Improved ACP Robot Fish [80]	◆ The lateral acceleration was measured with flow and pressure MEMS sensors and analyzed by video	➢ The force of the propulsion unit near the tail fin has increased by 31% and the frequency of fluctuations has doubled	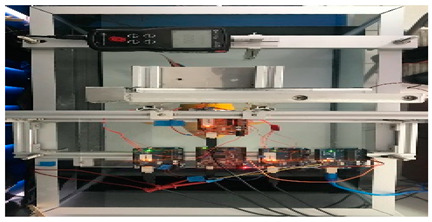
Four-link Carangiform Fish Robot [78]	◆ Free-swimming test under randomly set obstacles	➢ Overcame plane obstacles and semi-closed obstacles ➢ Determines the shortest path to the target area under the condition of obstacles	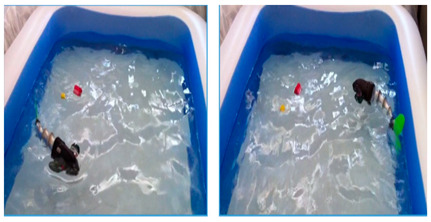
Four-link Robotic Fish Large Pectoral Fin Control Surfaces [73]	◆ Validation of the electromechanical design and swimming performance	➢ Robot fish realizes autonomous obstacle avoidance and three-dimensional swimming	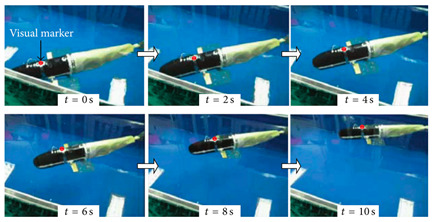
Fabricated Bionic Robotic Fish [70]	◆ The swimming speed and the angle of fin oscillation were measured	➢ Forward swimming velocity is 24.5 mm/s. ➢ The maximum force generated during fin oscillation is 0.39 N	N/A
ACP Robot Fish [81]	◆ Comparing locomotion properties with the kinematics	➢ Use materials with different bending stiffnesses for the compliant parts in different swimming models	N/A

**Table 11:** Thunniform biomimetic robots: experimental research.

Robot	Test Objectives	Test Conclusions	Picture
Gliding Robotic Dolphin [84]	◆ Carried out a hydrodynamic analysis of the gliding motion	➢ The robot can spiral and glide gently ➢ Obtained the hydrodynamic coefficients	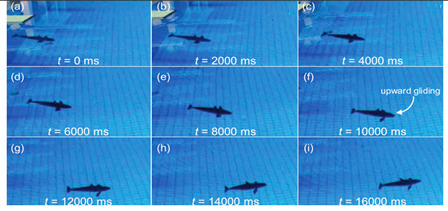
Single-Motor-Actuated Robotic Fish [85]	◆ The speed was measured when going straight and turning	➢ The maximum forward swimming speed is 1.14 m/s, and the speed is about 90 degrees/s during normal turns	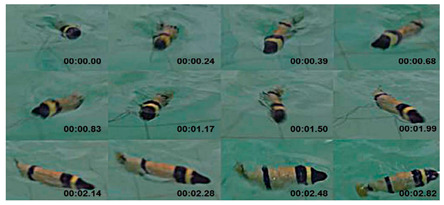
Thunniform Robotic Fish [43]	◆ Studied the relationship between the moving speed and the shock dynamics of the caudal fin	➢ The speed increases with the increase in the vibration frequency of the caudal fin, but there will be a certain threshold	N/A
Mackerel Robot [88]	◆ Analyzed the relationship between amplitude, efficiency and propulsion speed	➢ The optimal thrust efficiency is in the range of Strouhal number (St) 0.3 ≤ St ≤ 0.325	N/A

**Table 12:** Rajiform biomimetic robots: experimental research.

Robot	Test Objectives	Test Conclusions	Picture
Cartilage Structure Underwater Robot [95]	◆ Robots without cartilage and combined with soft cartilage materials were designed for experiments	➢ Incorporating cartilage structures into the fins improves swimming efficiency. A reasonable arrangement of soft and hard structures is important	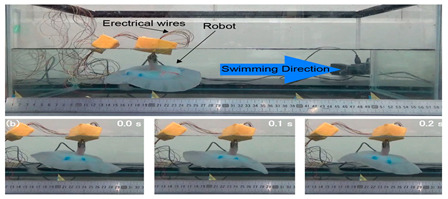
Manta Ray Robot [92]	◆ Tested the motion performance of the robot prototype	➢ Can realize simple pitch and roll motion patterns ➢ Can swim forward steadily	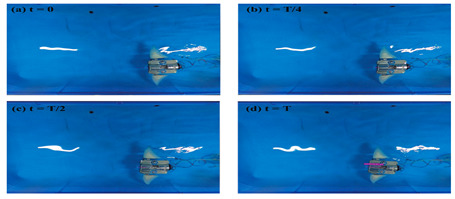
Self-Powered Soft Robot [90]	◆ Verify pressure resilience of electronics and soft actuators	➢ Activated in a field test at a depth of 10,900 m in the Mariana Trench ➢ Free swimming at a depth of 3224 m in the South China Sea ➢ The pressure resilience of the electronic components and soft actuators is reliable	N/A
IPMC Manta Ray [91]	◆ Analyzed the fins in terms of wing tip deflection, twist angle, and power consumption	➢ The swimming speed of the robot is 0.067 BL/s, and the mobile power consumption is below 2.5 W	N/A
Bionic Fin Manta Ray [93]	◆ Tested the motion performance of fins with different thicknesses	➢ The simple drive method of the design can produce a good propulsion effect	N/A

**Table 13:** Amiiform biomimetic robots: experimental research.

Robot	Test Objectives	Test Conclusions	Picture
RoboGnilos [98]	◆ Analyzed the influence of morphological parameters on undulating dynamics	➢ Verified the convenience and effectiveness of the modular motor drive structure	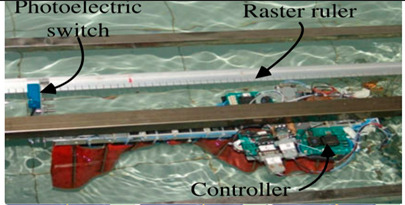
Bio-inspired Amphibious Robot [99]	◆ Tested the motion performance in water and on the ground	➢ Excellent undulating and flapping motion	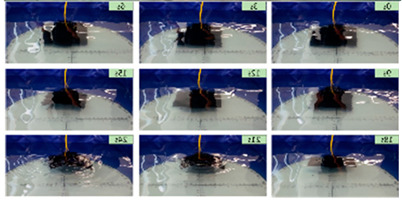
Dorsal Undulation Fin Robot [100]	◆ Experimental testing of robotic fish locomotor performance	➢ The proposed kinematic and dynamic models are effective for properties of undulating motion	N/A

**Table 14:** Gymnotiform biomimetic robots: experimental research.

Robot	Test Objectives	Test Conclusions	Picture
Undulatory Fin Propulsion Bio-Inspired Robot [103]	◆ Tested each movement mode of the robot ◆ Measured the swimming speed and azimuth angle	➢ Can complete forward swimming, reverse movement, diving, maintaining position and vertical swimming like a live fish	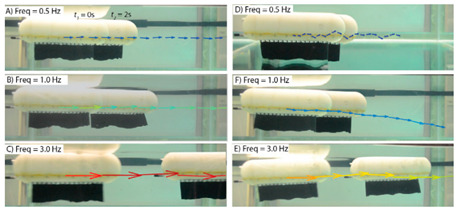
Robotic Knifefis [104]	◆ Determined how to decompose the force generated by a robotic fin into thrust and drag terms	➢ Measured surge and heave forces and swimming speed as a function of fin frequency, amplitude, and undulating number	N/A

## Data Availability

Data available within the article.

## References

[1] Isaka K., Tadami N., Fujiwara A., Watanabe T., Sugesawa M., Yamada Y., Yoshida H., Nakamura T. Study on Drilling Resistance Reduction of a Seafloor Robotic Explorer Based on the Drilling Properties of Underwater Ground. Proceedings of the 2019 IEEE/SICE International Symposium on System Integration (SII).

[2] Yamada D., Takebayashi T., Kato H., Sakagami N., Kawamura S. Underwater Robot with Negative Pressure Effect Plates for Maintenance of Underwater Structures. Proceedings of the 2019 IEEE/ASME International Conference On Advanced Intelligent Mechatronics (AIM).

[3] Ru J.Y., Yu H., Liu H., Liu J.Y., Zhang X.Y., Xu H.L. (2023). A Bounded Near-Bottom Cruise Trajectory Planning Algorithm for Underwater Vehicles. J. Mar. Sci. Eng..

[4] Leng D.X., Shao S., Xie Y.C., Wang H.H., Liu G.J. (2021). A brief review of recent progress on deep sea mining vehicle. Ocean. Eng..

[5] Zheng J.Z., Wang J.X., Guo X., Huntrakul C., Wang C., Xie G.M. (2022). Biomimetic Electric Sense-Based Localization: A Solution for Small Underwater Robots in a Large-Scale Environment. IEEE Robot. Autom. Mag..

[6] Colgate J.E., Lynch K.M. (2004). Mechanics and control of swimming: A review. IEEE J. Ocean. Eng..

[7] Bu K.L., Gong X.B., Yu C.L., Xie F. (2022). Biomimetic Aquatic Robots Based on Fluid-Driven Actuators: A Review. J. Mar. Sci. Eng..

[8] Chutia S., Kakoty N.M., Deka D. A Review of Underwater Robotics, Navigation, Sensing Techniques and Applications. Proceedings of the Advances in Robotics (AIR’17).

[9] Wang J., Wu Z.X., Dong H.J., Tan M., Yu J.Z. (2022). Development and Control of Underwater Gliding Robots: A Review. IEEE CAA J. Autom. Sin..

[10] Castano M.L., Tan X.B. (2019). Model Predictive Control-Based Path-Following for Tail-Actuated Robotic Fish. J. Dyn. Syst. Meas. Control. Trans. Asme.

[11] Ma Y.T., Ye R.D., Zheng R., Geng L.B., Yang Y. A highly mobile ducted underwater robot for subsea infrastructure inspection. Proceedings of the 2016 IEEE International Conference on Cyber Technology in Automation, Control, and Intelligent Systems (CYBER).

[12] Kruiper R., Vincent J.F.V., Abraham E., Soar R.C., Konstas I., Chen-Burger J., Desmulliez M.P.Y. (2018). Towards a Design Process for Computer-Aided Biomimetics. Biomimetics.

[13] Bianchi G., Maffi L., Tealdi M., Cinquemani S. (2023). A Bioinspired Cownose Ray Robot for Seabed Exploration. Biomimetics.

[14] Shao H., Dong B.B., Zheng C.Z., Li T., Zuo Q.Y., Xu Y.H., Fang H.T., He K., Xie F.R. (2022). Thrust Improvement of a Biomimetic Robotic Fish by Using a Deformable Caudal Fin. Biomimetics.

[15] Kocak M., Yazici M.V., Akdal E., Can F.C., Gezgin E. (2022). Utilization of Function Generation Synthesis on Biomimetics: A Case Study on Moray Eel Double Jaw Design. Biomimetics.

[16] Salazar R., Campos A., Fuentes V., Abdelkefi A. (2019). A review on the modeling, materials, and actuators of aquatic unmanned vehicles. Ocean. Eng..

[17] Silva A.T., Baerum K.M., Hedger R.D., Baktoft H., Fjeldstad H.P., Gjelland K.O., Okland F., Forseth T. (2020). The effects of hydrodynamics on the three-dimensional downstream migratory movement of Atlantic salmon. Sci. Total Environ..

[18] Triantafyllou M.S., Triantafyllou G.S. (1995). An efficient swimming machine. Sci. Am..

[19] Yu J.Z., Liu J.C., Wu Z.X., Fang H. (2018). Depth Control of a Bioinspired Robotic Dolphin Based on Sliding-Mode Fuzzy Control Method. IEEE Trans. Ind. Electron..

[20] Meng Y., Wu Z.X., Li Y.T., Chen D., Tan M., Yu J.Z. (2023). Vision-Based Underwater Target Following Control of an Agile Robotic Manta With Flexible Pectoral Fins. IEEE Robot. Autom. Lett..

[21] Yu J.Z., Wang C., Xie G.M. (2016). Coordination of Multiple Robotic Fish With Applications to Underwater Robot Competition. IEEE Trans. Ind. Electron..

[22] Yang Q., Li G., Mu W., Liu G., Sun H. (2020). Identification of crack length and angle at the center weld seam of offshore platforms using a neural network approach. J. Mar. Sci. Eng..

[23] Leng D.X., Liu D., Li H.Y., Jin B., Liu G.J. (2022). Internal flow effect on the cross-flow vortex-induced vibration of marine risers with different support methods. Ocean Eng..

[24] Szymak P., Praczyk T., Naus K., Szturomski B., Malec M., Morawski M. Research on Biomimetic Underwater Vehicles for Underwater ISR. Proceedings of the Ground/Air Multisensor Interoperability, Integration, and Networking for Persistent ISR VII.

[25] Wang R., Wang S., Wang Y., Cheng L., Tan M. (2022). Development and Motion Control of Biomimetic Underwater Robots: A Survey. IEEE Trans. Syst. Man Cybern. Syst..

[26] Wang A., Liu G., Wang X., Fu B. (2016). Development and Analysis of Body and/or Caudal Fin Biomimetic Robot Fish. J. Mech. Eng..

[27] Salazar R., Fuentes V., Abdelkefi A. (2018). Classification of biological and bioinspired aquatic systems: A review. Ocean Eng..

[28] Low K.H. (2009). Modelling and parametric study of modular undulating fin rays for fish robots. Mech. Mach. Theory.

[29] Li Y., Xu Y., Wu Z., Ma L., Guo M., Li Z., Li Y. (2022). A comprehensive review on fish-inspired robots. Int. J. Adv. Robot. Syst..

[30] Gafurov S.A., Klochkov E.V. Autonomous unmanned underwater vehicles development tendencies. Proceedings of the 2nd International Conference on Dynamics and Vibroacoustics of Machines (DVM2014).

[31] Raj A., Thakur A. (2016). Fish-inspired robots: Design, sensing, actuation, and autonomy—A review of research. Bioinspir. Biomim..

[32] Sfakiotakis M., Lane D.M., Davies J.B.C. (1999). Review of fish swimming modes for aquatic locomotion. IEEE J. Ocean. Eng..

[33] Liu G., Liu Z., Tian X., Wang Q., Chen G. (2018). A Review of the Application of Intelligent Materials in Underwater Biomimetic Robot. Period. Ocean Univ. China.

[34] Fu S.H., Wei F.N., Yin C., Yao L.G., Wang Y.X. (2021). Biomimetic soft micro-swimmers: From actuation mechanisms to applications. Biomed. Microdevices.

[35] Scaradozzi D., Palmieri G., Costa D., Pinelli A. (2017). BCF swimming locomotion for autonomous underwater robots: A review and a novel solution to improve control and efficiency. Ocean Eng..

[36] Lamas M.I., Rodriguez C.G. (2020). Hydrodynamics of Biomimetic Marine Propulsion and Trends in Computational Simulations. J. Mar. Sci. Eng..

[37] Zhou Z.Y., Liu J.C., Yu J.Z. (2022). A Survey of Underwater Multi-Robot Systems. IEEE-CAA J. Autom. Sin..

[38] Webb P.W. (1984). Form and function in fish swimming. Sci. Am..

[39] Lindsey C.C. (1978). Form, function and locomotory habits in fish. Fish Physiol..

[40] Wang C., Lu J., Ding X., Jiang C., Yang J., Shen J. (2021). Design, modeling, control, and experiments for a fish-robot-based IoT platform to enable smart ocean. IEEE Internet Things J..

[41] Guanrong H., Zhenlong W., Jian L.I., Yangwei W. (2008). Development of a Caudal-Fin-Propelled Micro Robot Fish Based on Flexible Fins. Robot.

[42] Butail S., Bartolini T., Porfiri M. (2013). Collective Response of Zebrafish Shoals to a Free-Swimming Robotic Fish. PLoS ONE.

[43] Mitin I., Korotaev R., Ermolaev A., Mironov V., Lobov S.A., Kazantsev V.B. (2022). Bioinspired Propulsion System for a Thunniform Robotic Fish. Biomimetics.

[44] Fish F.E. (1996). Transitions from drag-based to lift-based propulsion in mammalian swimming. Am. Zool..

[45] Rohr J.J., Fish F.E. (2004). Strouhal numbers and optimization of swimming by odontocete cetaceans. J. Exp. Biol..

[46] Wiguna T., Heo S., Park H.C., Goo N.S. (2009). Design and Experimental Parameteric Study of a Fish Robot Actuated by Piezoelectric Actuators. J. Intell. Mater. Syst. Struct..

[47] Edwards E.F. (2006). Duration of unassisted swimming activity for spotted dolphin (*Stenella attenuata*) calves: Implications for mother-calf separation during tuna purse-seine sets. Fish. Bull..

[48] Brill R.W. (1996). Selective advantages conferred by the high performance physiology of tunas, billfishes, and dolphin fish. Comp. Biochem. Physiol. A Physiol..

[49] Yu J.Z., Wu Z.X., Su Z.S., Wang T.Z., Qi S.W. (2019). Motion Control Strategies for a Repetitive Leaping Robotic Dolphin. IEEE-Asme Trans. Mechatron..

[50] Chan W.L., Kang T., Lee Y.J. Experiments and identification of an ostraciiform fish robot. Proceedings of the 2007 IEEE International Conference on Robotics and Biomimetics.

[51] Ikeda M., Hikasa S., Watanabe K., Nagai I. Propulsive force analysis of a pectoral fin for rajiform type fish robots from fluid dynamic aspects. Proceedings of the Eighteenth International Symposium on Artificial Life and Robotics (AROB 18th ‘13).

[52] Miyazaki H., Onoda A., Terada H., Nakajima M. (2017). Species Identification of Pufferfish Products Using RAPD Analysis. Food Hyg. Saf. Sci..

[53] Bogan S., Taverne L., Agnolin F. (2013). First Triassic and oldest record of a South American amiiform fish: Caturus sp from the Los Menucos Group (lower Upper Triassic), Rio Negro province, Argentina. Geol. Belg..

[54] Crampton W.G.R. (1996). Gymnotiform fish: An important component of Amazonian floodplain fish communities. J. Fish Biol..

[55] George A.B., Westneat M.W. (2021). Three-dimensional kinematic analyses reveal asymmetries in Xanthichthys auromarginatus (Balistidae) median fin biomechanics during steady balistiform swimming. Integr. Comp. Biol..

[56] Zhang R., Hu W. (2020). The numerical study on the propulsive mechanism of balistiform. Chin. J. Hydrodynomics.

[57] Chinook Salmon. Oncorhynchus Tshawytscha. http://www.nmfs.noaa.gov/pr/species/fish/chinook-salmon.html.

[58] Sagong W., Jeon W.-P., Choi H. (2013). Hydrodynamic characteristics of the sailfish (Istiophorus platypterus) and swordfish (Xiphias gladius) in gliding postures at their cruise speeds. PLoS ONE.

[59] Stingray. A-z Animals. https://a-z-animals.com/animals/stingray/?r.

[60] Gautreau E., Bonnet X., Sandoval J., Fosseries G., Herrel A., Arsicault M., Zeghloul S., Laribi M.A. (2022). A Biomimetic Method to Replicate the Natural Fluid Movements of Swimming Snakes to Design Aquatic Robots. Biomimetics.

[61] Crespi A., Ijspeert A.J. AmphiBot II: An amphibious snake robot that crawls and swims using a central pattern generator. Proceedings of the 9th International Conference on Climbing and Walking Robots (CLAWAR 2006).

[62] Stefanini C., Orofino S., Manfredi L., Mintchev S., Marrazza S., Assaf T., Capantini L., Sinibaldi E., Grillner S., Wallen P. A compliant bioinspired swimming robot with neuro-inspired control and autonomous behavior. Proceedings of the 2012 IEEE International Conference on Robotics and Automation.

[63] Liljebäck P., Stavdahl Ø., Pettersen K.Y., Gravdahl J.T. Mamba-A waterproof snake robot with tactile sensing. Proceedings of the 2014 IEEE/RSJ International Conference on Intelligent Robots and Systems.

[64] Lyu F., Xu X., Zha X., Li Z., Yuan H. A Snake Eel Inspired Multi-joint Underwater Inspection Robot for Undersea Infrastructure Intelligent Maintenance. Proceedings of the OCEANS 2022.

[65] Nguyen D.Q., Ho V.A. (2022). Anguilliform Swimming Performance of an Eel-Inspired Soft Robot. Soft Robot..

[66] Crespi A., Karakasiliotis K., Guignard A., Ijspeert A.J. (2013). Salamandra Robotica II: An Amphibious Robot to Study Salamander-Like Swimming and Walking Gaits. IEEE Trans. Robot..

[67] Liu J., Hu H. (2010). Biological inspiration: From carangiform fish to multi-joint robotic fish. J. Bionic Eng..

[68] Katzschmann R.K., Marchese A.D., Rus D. (2016). Hydraulic Autonomous Soft Robotic Fish for 3D Swimming. Experimental Robotics.

[69] Marchese A.D., Onal C.D., Rus D. (2014). Autonomous Soft Robotic Fish Capable of Escape Maneuvers Using Fluidic Elastomer Actuators. Soft Robot..

[70] Palani I.A., Muralidharan M. (2021). Development of Subcarangiform Bionic Robotic Fish Propelled by Shape Memory Alloy Actuators. Def. Sci. J..

[71] Clapham R.J., Hu H. iSplash-I: High performance swimming motion of a carangiform robotic fish with full-body coordination. Proceedings of the 2014 IEEE International Conference on Robotics and Automation (ICRA).

[72] Clapham R.J., Hu H. (2015). iSplash: Realizing fast carangiform swimming to outperform a real fish. Robot Fish: Bio-Inspired Fishlike Underwater Robots.

[73] Yu J., Wang K., Tan M., Zhang J. (2014). Design and control of an embedded vision guided robotic fish with multiple control surfaces. Sci. World J..

[74] Wiguna T., Heo S., Park H.C., Goo N.S. (2006). Mechanical Design of Biomimetic Fish Robot Using LIPCA as Artificial Muscle. Key Eng. Mater..

[75] Tong R., Wu Z., Chen D., Wang J., Du S., Tan M., Yu J. (2022). Design and Optimization of an Untethered High-Performance Robotic Tuna. IEEE/ASME Trans. Mechatron..

[76] Chen D., Wu Z., Meng Y., Tan M., Yu J. (2022). Development of a High-Speed Swimming Robot With the Capability of Fish-Like Leaping. IEEE/ASME Trans. Mechatron..

[77] Glaze J., Salazar R., Vasconcellos R., Abdelkefi A. (2021). Comparative design, hydrodynamic analysis, and physical performance of fish-like robots. Appl. Ocean Res..

[78] Ozmen Koca G., Korkmaz D., Bal C., Akpolat Z.H., Ay M. (2016). Implementations of the route planning scenarios for the autonomous robotic fish with the optimized propulsion mechanism. Measurement.

[79] Marchese A.D., Katzschmann R.K., Rus D. (2015). A Recipe for Soft Fluidic Elastomer Robots. Soft Robot.

[80] Von Borstel F.D., Haro M.S., Villa-Medina J.F., Gutiérrez J. (2022). Propulsive Element Normal Force Based on Acceleration Measurements Experienced by a Subcarangiform Robotic Fish. J. Intell. Robot. Syst..

[81] Zhong Y., Song J., Yu H., Du R. (2018). Toward a Transform Method From Lighthill Fish Swimming Model to Biomimetic Robot Fish. IEEE Robot. Autom. Lett..

[82] Zheng C.Z., Ding J., Dong B.B., Lian G.Y., He K., Xie F.R. (2022). How Non-Uniform Stiffness Affects the Propulsion Performance of a Biomimetic Robotic Fish. Biomimetics.

[83] Marras S., Porfiri M. (2012). Fish and robots swimming together: Attraction towards the robot demands biomimetic locomotion. J. R. Soc. Interface.

[84] Wu Z., Yu J., Yuan J., Tan M., Zhang J. Mechatronic design and implementation of a novel gliding robotic dolphin. Proceedings of the 2015 IEEE International Conference on Robotics and Biomimetics (ROBIO).

[85] Yu J., Zhang C., Liu L. (2016). Design and Control of a Single-Motor-Actuated Robotic Fish Capable of Fast Swimming and Maneuverability. IEEE/ASME Trans. Mechatron..

[86] Mainong A., Ayob A., Arshad M. (2017). Investigating pectoral shapes and locomotive strategies for conceptual designing bio-inspired robotic fish. J. Eng. Sci. Technol..

[87] Wang W., Xie G. (2014). CPG-based Locomotion Controller Design for a Boxfish-like Robot. Int. J. Adv. Robot. Syst..

[88] Wen L., Wang T., Wu G., Liang J. (2013). Quantitative Thrust Efficiency of a Self-Propulsive Robotic Fish: Experimental Method and Hydrodynamic Investigation. IEEE/ASME Trans. Mechatron..

[89] Crespi A., Lachat D., Pasquier A., Ijspeert A.J. (2007). Controlling swimming and crawling in a fish robot using a central pattern generator. Auton. Robot..

[90] Li G., Chen X., Zhou F., Liang Y., Xiao Y., Cao X., Zhang Z., Zhang M., Wu B., Yin S. (2021). Self-powered soft robot in the Mariana Trench. Nature.

[91] Chen Z., Um T.I., Bart-Smith H. (2012). Bio-inspired robotic manta ray powered by ionic polymer–metal composite artificial muscles. Int. J. Smart Nano Mater..

[92] Liu Q., Chen H., Wang Z., He Q., Chen L., Li W., Li R., Cui W. (2022). A Manta Ray Robot with Soft Material Based Flapping Wing. J. Mar. Sci. Eng..

[93] Chew C.-M., Lim Q.-Y., Yeo K. Development of propulsion mechanism for Robot Manta Ray. Proceedings of the 2015 IEEE International Conference on Robotics and Biomimetics (ROBIO).

[94] Valdivia y Alvarado P., Chin S., Larson W., Mazumdar A., Youcef-Toumi K. A soft body under-actuated approach to multi degree of freedom biomimetic robots: A stingray example. Proceedings of the 2010 3rd IEEE RAS & EMBS International Conference on Biomedical Robotics and Biomechatronics.

[95] Yurugi M., Shimanokami M., Nagai T., Shintake J., Ikemoto Y. (2021). Cartilage structure increases swimming efficiency of underwater robots. Sci. Rep..

[96] Huang H., Sheng C., Wu J., Wu G., Zhou C., Wang H. (2021). Hydrodynamic analysis and motion simulation of fin and propeller driven manta ray robot. Appl. Ocean Res..

[97] Zhou C., Low K.H. (2012). Design and Locomotion Control of a Biomimetic Underwater Vehicle With Fin Propulsion. IEEE/ASME Trans. Mechatron..

[98] Hu T., Shen L., Lin L., Xu H. (2009). Biological inspirations, kinematics modeling, mechanism design and experiments on an undulating robotic fin inspired by Gymnarchus niloticus. Mech. Mach. Theory.

[99] Yin S., Hu Q., Zeng Y., Wei C., Chen Z. Kinetic Analysis and Design of a Bio-Inspired Amphibious Robot with Two Undulatory Fins. Proceedings of the 2021 IEEE International Conference on Real-time Computing and Robotics (RCAR).

[100] Xie H., Zhou H., Shen L., Yin D. (2016). Mechanism Design, Dynamics Modelling and Experiments of Bionic Undulating Fins. Int. J. Robot. Autom..

[101] Low K., Zhou C., SEET G.G., Yu J. (2008). Learning from Gymnotiform swimmers—Design and implementation of robotic knifefish NkF-II. Int. J. Inf. Acquis..

[102] Pham C.A.T., Kim D.H., Nguyen T.T. A study on force generated by gymnotiform undulating fin. Proceedings of the 2018 15th International Conference on Ubiquitous Robots (UR).

[103] Liu H., Curet O. (2018). Swimming performance of a bio-inspired robotic vessel with undulating fin propulsion. Bioinspir. Biomim..

[104] Curet O.M., Patankar N.A., Lauder G.V., MacIver M.A. (2011). Mechanical properties of a bio-inspired robotic knifefish with an undulatory propulsor. Bioinspir. Biomim..

[105] Siahmansouri M., Ghanbari A., Fakhrabadi M.M.S. (2011). Design, implementation and control of a fish robot with undulating fins. Int. J. Adv. Robot. Syst..

[106] Zhang S., Qian Y., Liao P., Qin F., Yang J. (2016). Design and Control of an Agile Robotic Fish With Integrative Biomimetic Mechanisms. IEEE/ASME Trans. Mechatron..

[107] Behbahani S.B., Tan X. (2016). Bio-inspired flexible joints with passive feathering for robotic fish pectoral fins. Bioinspir. Biomim..

[108] Sitorus P.E., Nazaruddin Y.Y., Leksono E., Budiyono A. (2009). Design and Implementation of Paired Pectoral Fins Locomotion of Labriform Fish Applied to a Fish Robot. J. Bionic Eng..

[109] Gray J., Hancock G. (1955). The propulsion of sea-urchin spermatozoa. J. Exp. Biol..

[110] Taylor G.I. (1952). Analysis of the swimming of long and narrow animals. Proc. R. Soc. Lond. Ser. A. Math. Phys. Sci..

[111] Lighthill M. (1960). Note on the swimming of slender fish. J. Fluid Mech..

[112] Lighthill M.J. (1970). Aquatic animal propulsion of high hydromechanical efficiency. J. Fluid Mech..

[113] Wu T.Y.-T. (1971). Hydromechanics of swimming propulsion. Part 1. Swimming of a two-dimensional flexible plate at variable forward speeds in an inviscid fluid. J. Fluid Mech..

[114] Wu T.Y.-T. (1971). Hydromechanics of swimming propulsion. Part 2. Some optimum shape problems. J. Fluid Mech..

[115] Wu T.Y.-T. (1971). Hydromechanics of swimming propulsion. Part 3. Swimming and optimum movements of slender fish with side fins. J. Fluid Mech..

[116] Chopra M. (1974). Hydromechanics of lunate-tail swimming propulsion. J. Fluid Mech..

[117] Cheng J.-Y., Blickhan R. (1994). Bending moment distribution along swimming fish. J. Theor. Biol..

[118] Costello J.H., Colin S.P., Gemmell B.J., Dabiri J.O. (2019). Hydrodynamics of Vortex Generation during Bell Contraction by the Hydromedusa Eutonina indicans (Romanes, 1876). Biomimetics.

[119] Stamhuis E.J., Videler J.J. (1995). Quantitative flow analysis around aquatic animals using laser sheet particle image velocimetry. J. Exp. Biol..

[120] Triantafyllou M., Barrett D., Brown N., Morgan B., Yue D.P., Anderson J. (1996). A new paradigm of propulsion and maneuvering for marine vehicles. Discussion. Authors’ closure. Trans. -Soc. Nav. Archit. Mar. Eng..

[121] Costa D., Palmieri G., Palpacelli M.C., Scaradozzi D., Callegari M. (2020). Design of a Carangiform Swimming Robot through a Multiphysics Simulation Environment. Biomimetics.

[122] Lau W.P., Zhong Y., Du R., Li Z. Bladderless swaying wire-driven robot shark. Proceedings of the 2015 IEEE 7th International Conference on Cybernetics and Intelligent Systems (CIS) and IEEE Conference on Robotics, Automation and Mechatronics (RAM).

[123] Zhao W., Hu Y., Wang L., Zhang L. (2009). Design and CPG-based control of biomimetic robotic fish. IET Control Theory Appl..

